# LOC496300 is expressed in the endoderm of developing *Xenopus laevis* embryos

**DOI:** 10.17912/micropub.biology.000150

**Published:** 2019-08-12

**Authors:** Maria E Stewart, Kelsey M Donahue, Elizabeth G Wilke, Emily T Shifley

**Affiliations:** 1 Northern Kentucky University; Highland Heights, KY

**Figure 1.**
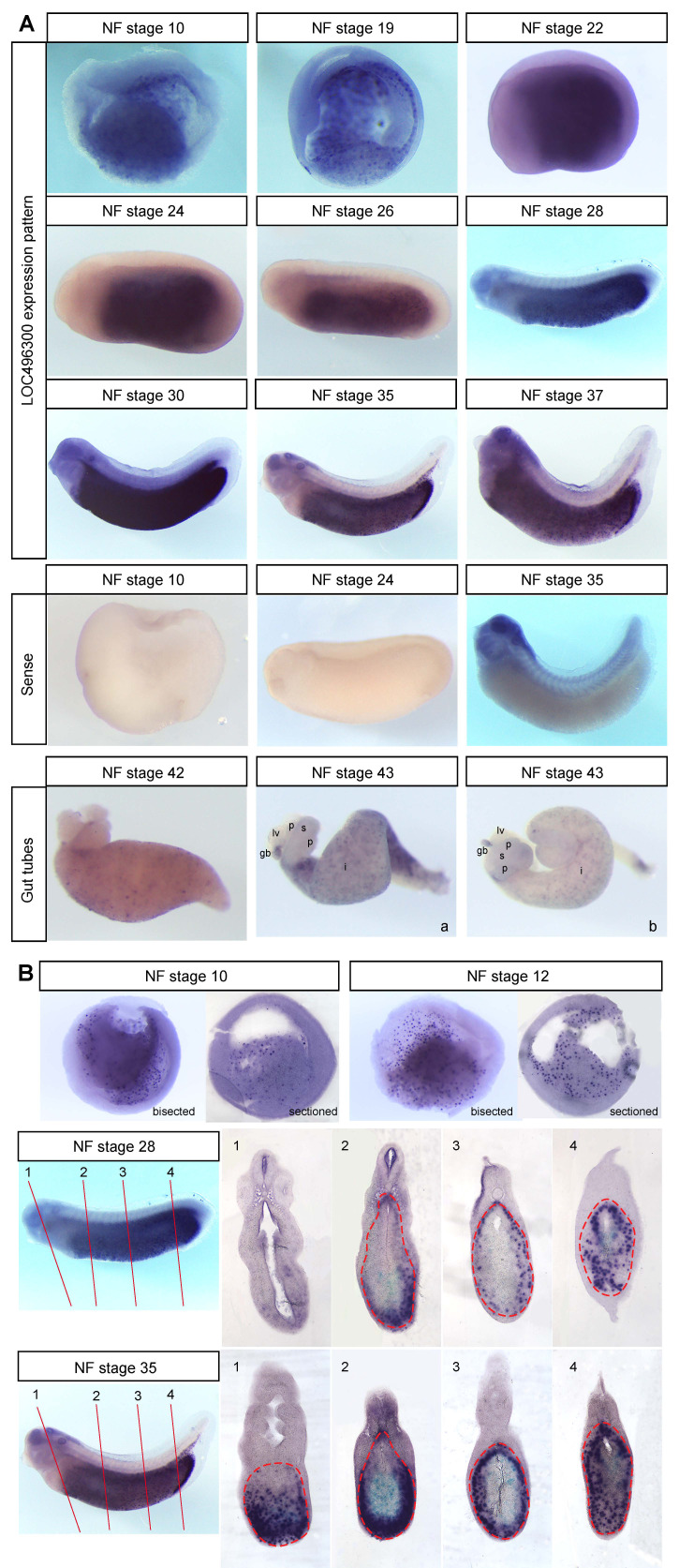
**Figure 1. (A)** LOC496300 is expressed in the endoderm of *Xenopus laevis* from NF stage 10 through 37. It becomes restricted to the midgut and hindgut during tailbud stages (~NF stg 30) and is expressed in the intestine and gall bladder of NF stage 42-43 gut tubes at differing levels (i.e. gut tube a and b). Embryos hybridized with the sense probe for LOC496300 show some non-specific staining in the head at tailbud stages, but overall no specific staining in the endoderm. NF stage 43 organ buds are marked as follows: lv, liver; p, pancreas; s, stomach; gb, gall bladder; i, intestine. **(B)** NF stage 10, 12, 28 and 35 embryos were sectioned to show the punctate expression pattern of LOC496300 throughout the early endoderm and in the dorsal and ventral tailbud endoderm. The numbered lines mark the approximate level of each section of the tailbud embryos and the dotted red line outlines the endoderm.

## Description

*Xenopus laevis* is an excellent model organism for studies on vertebrate endoderm development (Womble *et al*., 2016; Zorn, 2009). The endoderm contributes to a number of important organs including the lungs, liver, gall bladder, pancreas, stomach and intestine. Each of these organs differentiate from the foregut, midgut, and hindgut domains of the early endoderm (Chalmers and Slack, 2000). It has recently been shown that as early as gastrulation differential fates are being specified within the endoderm (Costa *et al*., 2003; Rankin *et al*., 2018). Several studies have worked to identify markers of different fates in the developing endoderm in order to assist with research on endoderm specification, differentiation, and organogenesis, but not all of the identified genes have known functions and some are currently unclassified (Chen *et al*., 2003; Costa *et al*., 2003; Park *et al*., 2007; Zorn and Mason 2001).

We performed whole mount *in situ* hybridization (WISH) on hypothetical locus LOC496300 (Xl.8755; Ref Seq NM_001095458.1) and found that it is expressed throughout the endoderm of early *Xenopus* embryos (NF stage 10-23) and as development proceeds it becomes localized to the midgut and hindgut endoderm (NF stage 27-37) (Figure 1A), which later gives rise to the intestine posterior to the stomach (Chalmers and Slack, 1998). LOC496300 has a similar expression pattern to other midgut-hindgut specific markers like *ctbs*, *impdh1*, and *darmin* during the early tadpole stages (Costa *et al*., 2003; Pera *et al*., 2003). LOC496300 does show some expression in the foregut early on, whereas *darmin* is excluded from the foregut even at gastrulation (Costa *et al*., 2003, Pera *et al*., 2003). We repeated the WISH for LOC496300 with three sets of embryos and saw consistent expression patterns with each set (examining a total of at least 18 embryos for each stage). WISH with the sense probe for LOC496300 as a control showed no staining in the endoderm at any stage, but non-specific staining in the brain, eye, and pharynx at NF stage 35 (see Figure 1A).

Sectioned embryos show expression of LOC496300 in the endoderm is punctate in a subset of cells throughout all regions of the early endoderm (Figure 1B). At tailbud stages LOC496300 is expressed in both the dorsal and ventral midgut and hindgut and in the gut tube at NF stage 42 and 43, maintaining a punctate expression pattern throughout the intestine. It is not clear what subset of cells might be expressing LOC496300. There are several other genes that show punctate expression patterns in the endoderm such as *Sox17α*, *Sox17β*, and *darmin* at NF stage 10 when there are many yolk granules still present in the endoderm cells (Costa *et al*., 2003; Hudson *et al*., 1997; Sinner *et al*., 2004). Later in development, it is possible LOC496300 is expressed in a group of cells that will differentiate into a specific cell type for the functional intestinal epithelium of the feeding tadpole, which consists mainly of principal absorptive cells as well as some gland cells, endocrine cells and lymphocytes (Marshall and Dixon; 1978; Shi and Ishizuya-Oka, 1996). This larval epithelium will eventually undergo apoptosis except for a few cells that de-differentiate into progenitor stem cells which give rise to the adult intestinal epithelium at metamorphosis (Ishizuya-Oka, 1996; Ishizuya-Oka, 2011; Ishizuya-Oka *et al*., 2009; Shi and Ishizuya-Oka, 1996; Sun *et al*., 2013). It would be interesting to determine which genes expressed in the embryonic intestine, like LOC496300, play a role in larval intestinal development and function, and for the dramatic transition that occurs during metamorphosis.

LOC496300 is currently unclassified. Searching for conserved domains identified a Spc7 kinetochore domain in LOC496300, suggesting it may play a role in kinetochore function. It also has KOG2044 and SbcC exonuclease domains, suggesting a possible role in replication, recombination, or repair. Further research into the functions of LOC496300 will help identify the role it plays specifically in the endoderm during development and whether it contributes to the specification and differentiation of the intestinal fate. Additionally, it would be interesting to discover whether LOC496300 is regulated by dynamic Wnt, BMP, and RA signals during different stages of development, as others have shown that coordination of these signaling pathways specifies different endodermal fates (Rankin *et al*., 2018; Stevens *et al*., 2017).

## Reagents

*Xenopus laevis* embryos were cultured and gut tubes were isolated using standard protocols approved by the NKU Institutional Animal Care and Use Committee (IACUC Protocol #2014-09). The plasmid to make the WISH probe for LOC496300 was purchased sequence-verified from Source Bioscience (CF286219 clone #IRBHp990A1273D) and we used SalI to linearize the plasmid and T7 to transcribe the anti-sense RNA probe (NotI with SP6 for the sense probe). We followed previously described protocols for WISH (Costa et al., 2003). For sectioning, the embryos were embedded in gelatin-albumin and cut into 50uM sections using a Leica vibratome VT1200 S.
